# Presynaptic Selectivity of a Ligand for Serotonin 1A Receptors Revealed by *In Vivo* PET Assays of Rat Brain

**DOI:** 10.1371/journal.pone.0042589

**Published:** 2012-08-07

**Authors:** Takeaki Saijo, Jun Maeda, Takashi Okauchi, Jun-ichi Maeda, Yasunori Morio, Yasuhiro Kuwahara, Masayuki Suzuki, Nobuharu Goto, Toshimitsu Fukumura, Tetsuya Suhara, Makoto Higuchi

**Affiliations:** 1 Molecular Imaging Center, National Institute of Radiological Sciences, Chiba, Japan; 2 Department of Molecular Neuroimaging, Tohoku University School of Medicine, Sendai, Japan; 3 DMPK Research Laboratory, Mitsubishi Tanabe Pharma Corporation, Kisarazu, Japan; 4 Research Quality Assurance Department, Mitsubishi Tanabe Pharma Corporation, Yokohama, Japan; 5 Advanced Medical Research Laboratory, Mitsubishi Tanabe Pharma Corporation, Yokohama, Japan; 6 Development Project Management Department, Mitsubishi Tanabe Pharma Corporation, Tokyo, Japan; 7 Early Stage Clinical Research Center, Mitsubishi Tanabe Pharma Corporation, Tokyo, Japan; RIKEN Brain Science Institution, Japan

## Abstract

A novel investigational antidepressant with high affinity for the serotonin transporter and the serotonin 1A (5-HT_1A_) receptor, called Wf-516 (structural formula: (2S)-1-[4-(3,4-dichlorophenyl)piperidin-1-yl]-3-[2-(5-methyl-1,3,4-oxadiazol-2-yl)benzo[b]furan-4-yloxy]propan-2-ol monohydrochloride), has been found to exert a rapid therapeutic effect, although the mechanistic basis for this potential advantage remains undetermined. We comparatively investigated the pharmacokinetics and pharmacodynamics of Wf-516 and pindolol by positron emission tomographic (PET) and autoradiographic assays of rat brains in order to elucidate their molecular interactions with presynaptic and postsynaptic 5-HT_1A_ receptors. In contrast to the full receptor occupancy by pindolol in PET measurements, the binding of Wf-516 to 5-HT_1A_ receptors displayed limited capacity, with relatively high receptor occupancy being achieved in regions predominantly containing presynaptic receptors. This selectivity was further proven by PET scans of neurotoxicant-treated rats deficient in presynaptic 5-HT_1A_ receptors. In addition, [^35^S]guanosine 5′-O-[γ-thio]triphosphate autoradiography indicated a partial agonistic ability of Wf-516 for 5-HT_1A_ receptors. This finding has lent support to reports that diverse partial agonists for 5-HT_1A_ receptors exert high sensitivity for presynaptic components. Thus, the present PET data suggest a relatively high capacity of presynaptic binding sites for partial agonists. Since our *in vitro* and *ex vivo* autoradiographies failed to illustrate these distinct features of Wf-516, *in vivo* PET imaging is considered to be, thus far, the sole method capable of pharmacokinetically demonstrating the unique actions of Wf-516 and similar new-generation antidepressants.

## Introduction

Selective serotonin reuptake inhibitors (SSRIs), such as fluvoxamine, fluoxetine and paroxetine, are currently the most frequently prescribed antidepressants [1]–[3]. SSRIs induce fewer adverse effects than classical tricyclic agents [4], thereby contributing to improved quality of life. These drugs increase serotonin (5-HT) concentration at synaptic clefts through inhibitory binding to 5-HT transporters (5-HTTs) responsible for 5-HT reuptake, thus enhancing serotonergic neurotransmissions and producing an antidepressant effect [5]. However, this serotonergic reinforcement does not take place immediately after the initiation of treatment, as increased 5-HT stimulates 5-HT 1A (5-HT_1A_) autoreceptors as negative feedback, inhibiting the release of 5-HT at presynaptic terminals [6], [7]. The persistent rise of 5-HT levels following repeated SSRI administration subsequently induces desensitization of 5-HT_1A_ autoreceptors, and the firing frequencies of 5-HT neurons gradually recover [8], [9], resulting in the delayed appearance of antidepressant effects. In practice, this delayed therapeutic benefit of SSRIs has been a source of distress for both depressive patients and psychiatrists.

Pindolol, a therapeutic agent used for the treatment of hypertension, antagonistically binds to not only β adrenergic receptors but also to central 5-HT_1A_ receptors [10], and its antagonism for 5-HT_1A_ receptors is assumed to interrupt the autoreceptor-mediated negative feedback. To date, several clinical trials have demonstrated that pindolol accelerates the alleviation of depressive symptoms following initiation of SSRI treatment [11]–[14]. For this serotonergic modulation, it is necessary for pindolol to preferentially block presynaptic 5-HT_1A_ autoreceptors without profound suppression of postsynaptic receptors, since postsynaptic antagonism could counteract the indirect agonism by SSRIs [10]. This selective binding property of pindolol for 5-HT_1A_ autoreceptors has been investigated using positron emission tomography (PET) with [^11^C]N-[2-[4-(2-methoxyphenyl)-1-piperazinyl]ethyl]-N-(2-pyridinyl)cyclohexane-carboxamide] ([^11^C]WAY-100635), a specific radioligand suitable for PET imaging of 5-HT_1A_ receptors [15], thus enabling quantification of occupancies of these receptors by therapeutic agents. Several reports have supported the selectivity of pindolol for presynaptic receptors abundantly located in the pontine raphe nucleus [16], [17], although such a binding preference has not been confirmed by other studies [18]. In a non-clinical PET study using [^11^C]WAY-100635, preferential binding of pindolol to 5-HT_1A_ autoreceptors was observed [19], but this was inconsistent with the findings of an *ex vivo* autoradiography (ARG) study that used intravenous administration of [^3^H]WAY-100635 and identified nonselective binding of pindolol to 5-HT_1A_ receptors in rat brain [20].

**Figure 1:**
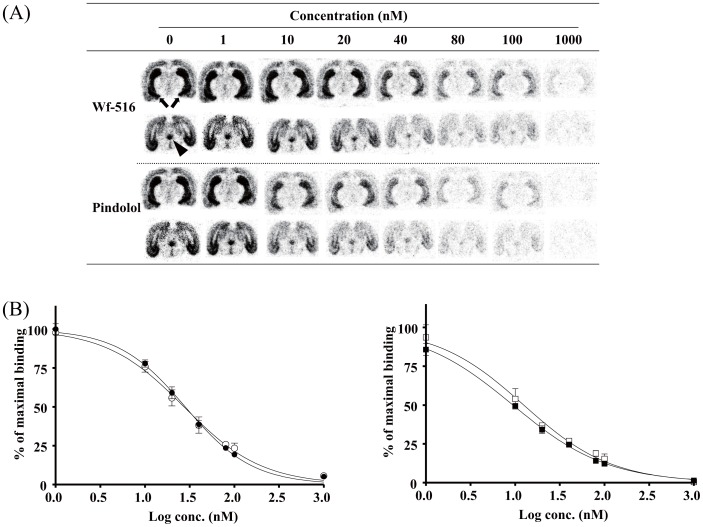
Binding affinities of Wf-516 and pindolol for central 5-HT_1A_ receptors quantified by *in vitro* ARG. (A) Representative autoradiograms showing distribution and intensity of [^11^C]WAY-100635 radiosignals in rat brain sections containing the hippocampus (arrows) and raphe nucleus (arrowhead) and its attenuation by different concentrations of Wf-516 or pindolol. (B) Inhibition curves of [^11^C]WAY-100635 binding to the hippocampus (closed symbols) and raphe nucleus (open symbols) by Wf-516 (left) and pindolol (right). Bars indicate S.E. (n = 3).

Wf-516 (structural formula: (2 S)-1-[4-(3,4-dichlorophenyl)piperidin-1-yl]-3-[2-(5-methyl-1,3,4-oxadiazol-2-yl)benzo[b]furan-4-yloxy]propan-2-ol monohydrochloride), a novel investigational antidepressant with high affinity for 5-HTT and 5-HT_1A_ receptors [21], has been shown to have more rapid antidepressant-like effects than the classical tricyclic antidepressant imipramine in a rat chronic mild stress model of depression [22]. Moreover, a recent *in vivo* electrophysiological study using rats has indicated that Wf-516 at low and medium doses was an antagonist for presynaptic but not postsynaptic 5-HT_1A_ receptors [23]. Although our previous PET study of rats demonstrated *in vivo* binding of Wf-516 to central 5-HTTs in a dose-dependent manner [24], the pharmacological mechanisms involved in the presynaptic/postsynaptic selectivity of binding of Wf-516 to 5-HT_1A_ receptors in living brains had still remained to be clarified using neuroimaging assays.

**Figure 2:**
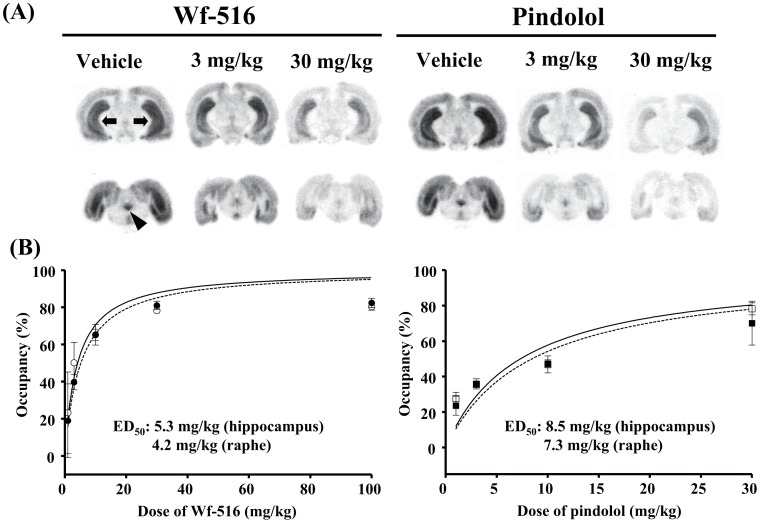
Occupancies of 5-HT_1A_ receptors by Wf-516 and pindolol assessed by *ex vivo* **ARG.** (A) Representative autoradiograms showing distribution and intensity of [^11^C]WAY-100635 radiosignals in rat brain sections containing the hippocampus (arrows) and raphe nucleus (arrowhead) after oral administration of Wf-516 and intraperitoneal administration of pindolol. (B) Relationships between dose of Wf-516 (left) or pindolol (right) and 5-HT_1A_ receptor occupancy in the hippocampus (closed symbols) and raphe nucleus (open symbols). Regression curves in the hippocampus and raphe nucleus are denoted by dashed lines and solid lines, respectively, and were generated by the following equation: Occ  = 100×D/(D+ED_50_), where Occ is 5-HT_1A_ receptor occupancy and D is the dose of the drug. Bars indicate S.E. (n = 3).

The present study was conducted in order to determine the properties of the interaction between Wf-516 and presynaptic and postsynaptic 5-HT_1A_ receptors localized predominantly in the raphe nucleus and hippocampus, respectively. Occupancies of these receptors by Wf-516 and pindolol were measured and compared in rat brains using PET with [^11^C]WAY-100635, and presynaptic selectivity of Wf-516 was further examined by treating rats with a toxin for 5-HT neurons.

**Figure 3:**
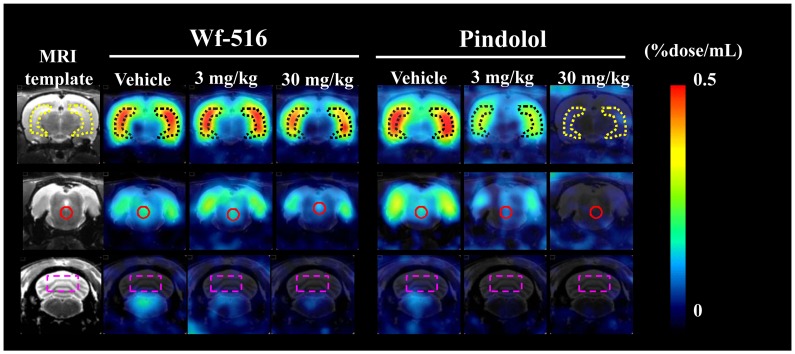
Representative PET images showing distribution of [^11^C]WAY-100635 in rat brains after oral administration of Wf-516 and intraperitoneal administration of pindolol. Each pretreatment drug was repeatedly administered to the same rat at different doses. PET images were generated by summation of dynamic data 60–90 min after intravenous injection of [^11^C]WAY-100635, and were overlaid on the MRI template displayed in the far left column. Coronal brain sections shown here were obtained at −5.2 mm (top row), −7.8 mm (middle row) and −12.5 mm (bottom row) from the bregma. ROIs were placed on the hippocampus (dotted lines), raphe nucleus (solid lines) and cerebellum (dashed lines).

## Methods

### Drugs and Chemicals

Wf-516 was synthesized at Mitsubishi Tanabe Pharma Co. (Osaka, Japan). Pindolol in a racemate form, fluvoxamine, 5,7-dihydroxytryptamine (DHT), WAY-100635, 8-hydroxy-2-(di-n-propylamino) tetralin (8-OH-DPAT), (±)-Metoprolol, guanosine diphosphate (GDP) and guanosine-5′-O-(3-thio)-triphosphate (GTPγS) were obtained from Sigma-Aldrich (St Louis, MO). Hydroxypropylmethyl cellulose (HPMC), desipramine hydrochloride, pentobarbital and [^35^S]GTPγS (47.46 TBq/mmol) were purchased from Shin-Etsu Chemical (Tokyo, Japan), Research Biochemicals International (Natick, MA), Dainippon Sumitomo Pharma (Osaka, Japan) and Perkin Elmer (Waltham, MA), respectively. Wf-516 was dissolved or suspended in 0.5% HPMC, and pindolol was dissolved in saline containing 0.1 M citrate when administered to rats. All other chemicals were of analytical grade and commercially available.

**Figure 4:**
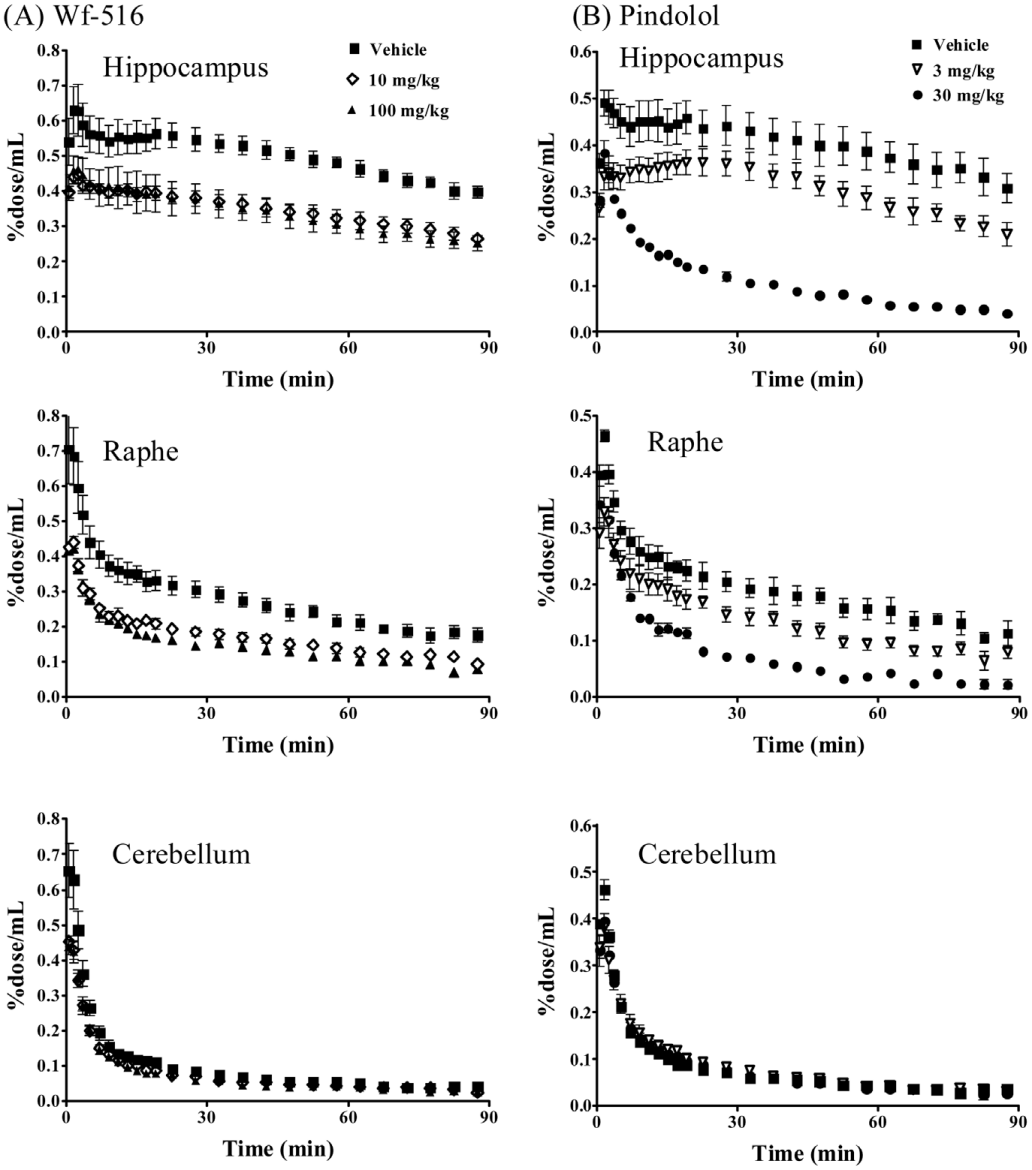
Time-radioactivity curves for [^11^C]WAY-100635 in the hippocampus (top panels), raphe nucleus (middle panels) and cerebellum (bottom panels) after pretreatments with Wf-516 (A) and pindolol (B) at representative doses. Data were generated by placing ROIs on different brain structures on the PET images illustrated in Figure 3. Radiotracer uptake into each region was expressed as a percentage of the injected dose per unit tissue volume (%dose/mL). Bars indicate S.E. (n = 4 and 3 in Wf-516 and pindolol treatment groups, respectively).

### Animals

The research protocols in the present study were approved by the Animal Ethics Committee of the National Institute of Radiological Sciences. Male Wistar rats at 8 weeks of age were purchased from CLEA Japan Inc. (Tokyo, Japan). Prior to the PET measurements, neuroanatomical template magnetic resonance images (MRI) of the rat brains were generated as described elsewhere [24].

**Figure 5:**
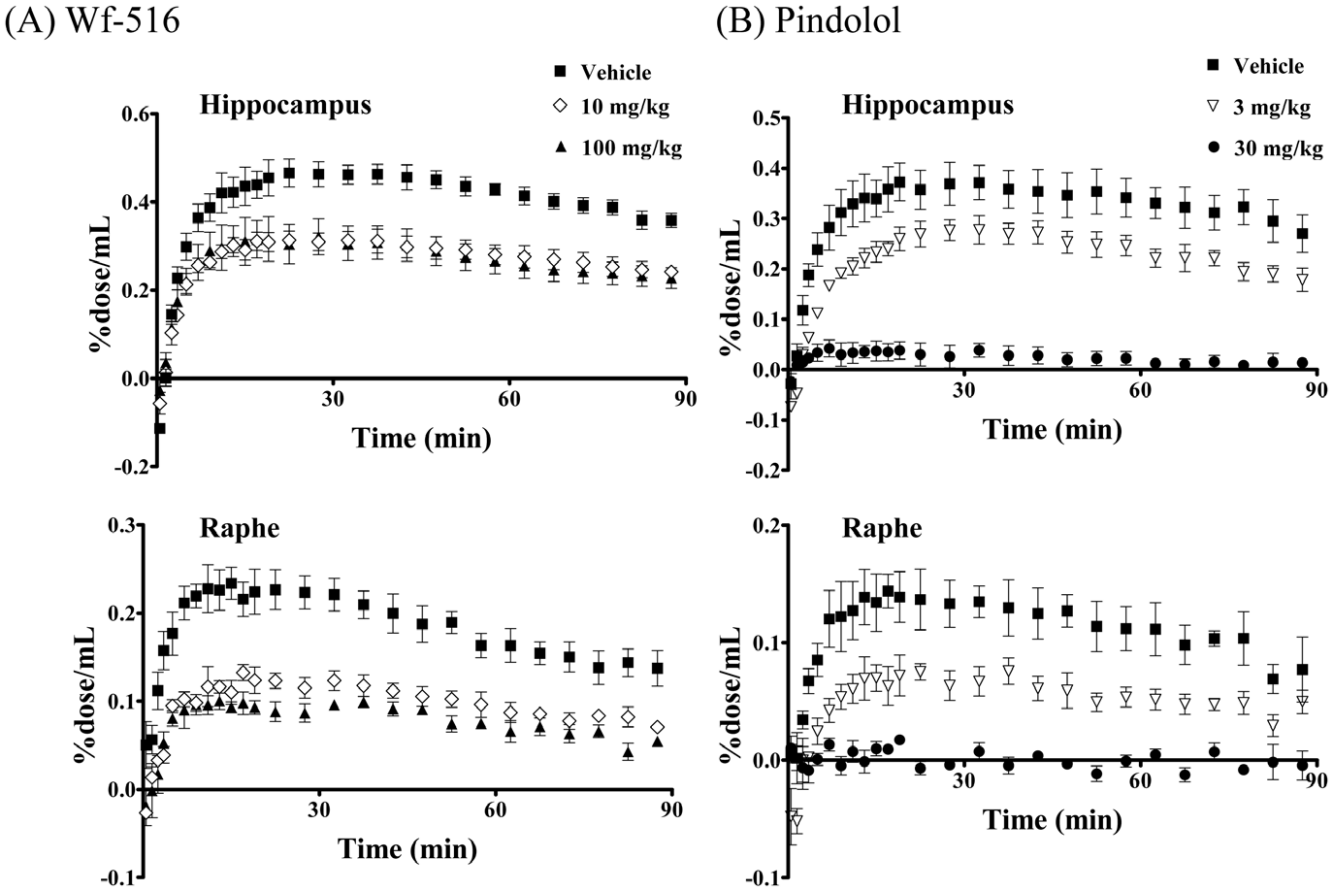
Time course of specific [^11^C]WAY-100635 binding to 5-HT_1A_ receptors in the hippocampus (top panels) and raphe nucleus (bottom panels) after pretreatments with Wf-516 (A) and pindolol (B) at representative doses. Data were generated by placing ROIs on different brain structures on the PET images illustrated in Figure 3. Binding was estimated as the difference in radiosignals between target and cerebellar regions, and expressed as the percentage of the injected dose per unit tissue volume (%dose/mL). Bars indicate S.E. (n = 4 and 3 in Wf-516 and pindolol treatment groups, respectively).

### Radioligand Synthesis

[^11^C]WAY-100635 was prepared by ^11^C-acylation of WAY-100634 with ^11^C-cyclohexanecarbonyl chloride as previously described [25]. Radiochemical purity of the radioligand was more than 95%, and specific radioactivity at the end of radiosynthesis was 183±67 GBq/µmol.

**Table 1:** BP_ND_ of [^11^C]WAY-100635 and occupancy of 5-HT_1A_ receptors by Wf-516 and pindolol.

			BP_ND_		Occupancy (%)	
	PET ligand	Dose (mg/kg)	Hippocampus	Raphe	Hippocampus	Raphe
Wf-516	[^11^C]WAY-100635	0	6.037±0.338	2.348±0.101	–	–
		1	5.612±0.356	2.128±0.109	7.0±2.7	8.5±7.6
		3	5.589±0.410	2.024±0.097	6.8±7.1	13.2±6.1
		10	4.937±0.550	1.620±0.126	18.8±6.0	30.6±6.4
		30	4.836±0.441	1.271±0.194	20.3±4.1	46.3±6.9
		100	4.962±0.400	1.212±0.073	17.8±4.9	48.2±3.3
	[^18^F]MPPF	0	1.433±0.051	0.418±0.051	–	–
		30	1.100±0.015	0.227±0.027	23.3±1.5	45.7±6.5
Pindolol	[^11^C]WAY-100635	0	5.709±0.724	1.798±0.316	–	–
		1	4.787±0.676	1.129±0.164	15.0±11.8	33.7±14.5
		3	3.517±0.119	0.752±0.093	37.0±5.6	57.3±2.8
		10	2.190±0.757	0.459±0.153	58.1±18.4	69.6±14.6
		30	0.441±0.173	0.029±0.029	91.8±3.4	98.3±1.7
		3+ FLV*	3.670±0.315	0.783±0.178	33.0±18.8	53.1±15.1
		30+FLV*	0.343±0.065	0	91.6±1.2	100.0

Data represent mean ± S.E. of 3–4 rats.

* Thirty mg/kg of fluvoxamine (FLV) was orally administered 30 min before pindolol treatment.

### Measurement of Ki Values of Wf-516 and Pindolol for 5-HT_1A_ Receptors by *In vitro* ARG

Rats were sacrificed by decapitation. Twenty-micrometer-thick frozen brain sections were preincubated for 30 min in 50 mM Tris-HCl (pH 7.4) containing 1 mM MnCl_2_. The samples were then incubated at room temperature for 60 min in the same buffer containing 0.9 nM [^11^C]WAY-100635 and either Wf-516 or pindolol at eight different concentrations (0–1000 nM). Nonspecific binding of the radioligand was estimated using 10 µM nonradioactive WAY-100635. Subsequently, the sections were rinsed twice with ice-cold Tris-HCl buffer for 5 min and then desalted with ice-cold distilled water for 10 sec. These samples were warmly blow-dried and placed in contact with an imaging plate (Fuji Film, Tokyo, Japan) for 60 min. Signals on the imaging plate were detected by BAS5000 system (Fuji Film). The inhibition constants of Wf-516 and pindolol for [^11^C]WAY-100635 (Ki values) were calculated using the following equation:
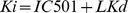
where IC_50_, L and Kd are median inhibitory concentration, radioligand concentration (0.9 nM) and dissociation constant of WAY-100635 (0.37 nM) [26], respectively.

**Figure 6:**
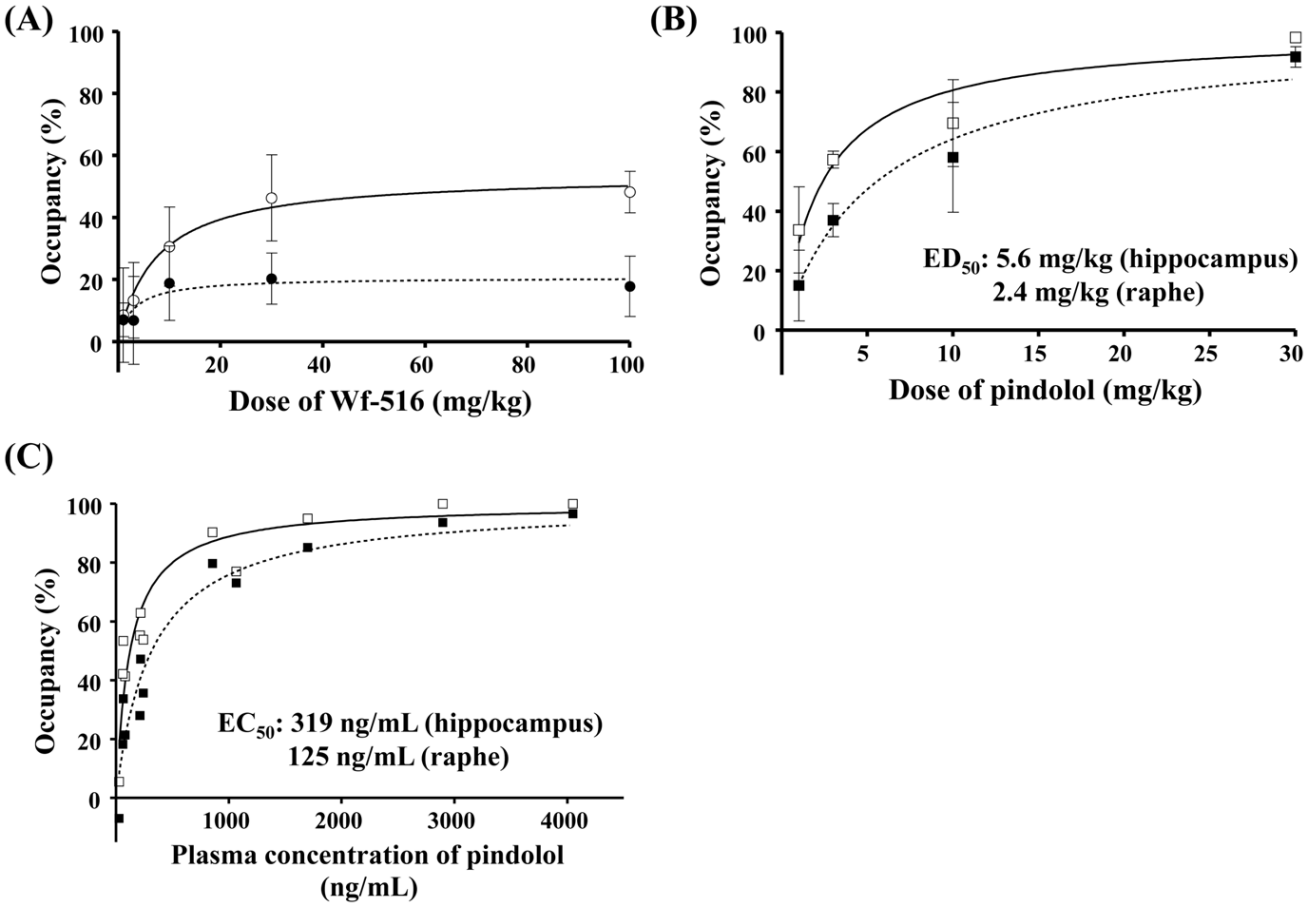
Relationships between dose or plasma concentration of test drugs and 5-HT_1A_ receptor occupancies analyzed with [^11^C]WAY-100635-PET data. (A) Receptor occupancies in the hippocampus (closed circles) and raphe nucleus (open circles) plotted against oral dose of Wf-516. Regression curves were generated by the following equation: Occ  = Occ_max_×D/(D+ED_50_), where Occ, Occ_max_, D and ED_50_ are 5-HT_1A_ receptor occupancy, maximal occupancy, dose of Wf-516, and dose of Wf-516 required for 50% of maximal occupancy, respectively. Bars indicate S.E. (n = 4). (B) Receptor occupancies in the hippocampus (closed squares) and raphe nucleus (open squares) plotted against intraperitoneal dose of pindolol. Regression curves were generated by the following equation: Occ  = 100×D/(D+ED_50_). Bars indicate S.E. (n = 3). (C) Receptor occupancies in the hippocampus (closed squares) and raphe nucleus (open squares) plotted against plasma concentration of pindolol. Regression curves were generated by the following equation: Occ  = 100×C/(C+EC_50_), where C is plasma concentration of pindolol. Dashed and solid lines represent regressions in the hippocampus and raphe nucleus, respectively.

### Measurement of 5-HT_1A_ Receptor Occupancy by Drugs Using *Ex vivo* ARG

Wf-516 (vehicle only, 1, 3, 10, 30 and 100 mg/kg) and pindolol (vehicle only, 1, 3, 10 and 30 mg/kg) were orally and intraperitoneally administered to rats, respectively. Animals were then killed by decapitation 5 h and 30 min after their administration, respectively. Autoradiographic analysis was performed using procedures similar to *in vitro* ARG except for incubation temperature (37°C) and times for preincubation (30 sec), incubation (5 min) and rinsing (1 min). Occupancy of 5-HT_1A_ receptors by Wf-516 and pindolol was calculated as the drug-induced reduction of [^11^C]WAY-100635 binding relative to vehicle-treated controls in each region of interest (ROI). The doses of these drugs at half-maximal effect (ED_50_) were determined according to the following relationship:
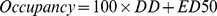
where D is the dose of the drug.

**Figure 7:**
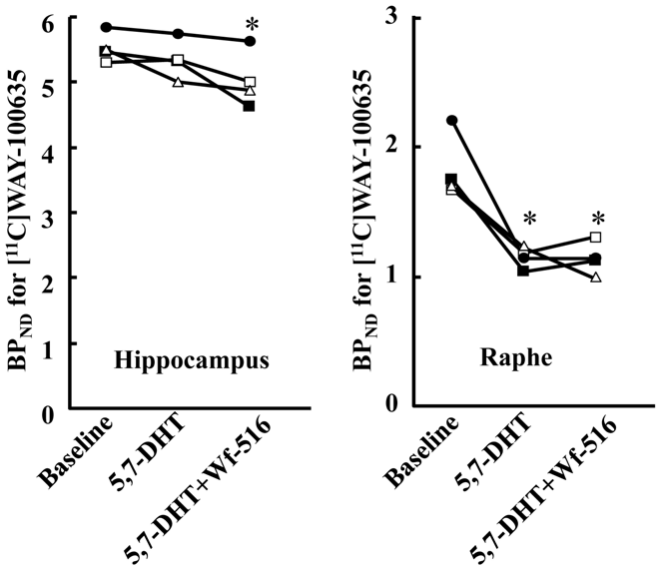
Alteration of BP_ND_ for [^11^C]WAY-100635 in rats treated with a toxicant for 5-HT neurons, 5,7-DHT. Four rats each underwent three [^11^C]WAY-100635-PET scans, at baseline, after 5,7-DHT treatment, and after oral administration of 30 mg/kg Wf-516 (5,7-DHT + Wf-516), in the indicated chronological order. Each symbol represents individual BP_ND_ in the hippocampus (left) and raphe nucleus (right). These changes of BP_ND_ in each region were statistically examined by one-way repeated-measures ANOVA followed by least significant difference test. *p<0.05 compared with each baseline.

### Measurement of 5-HT_1A_ Receptor Occupancy by Drugs Using [^11^C]WAY-100635-PET

A series of 6 and 5 dynamic PET scans was performed for each rat approximately 5 h and 30 min after oral and intraperitoneal pretreatments with graded doses of Wf-516 (vehicle only, 1, 3, 10, 30 and 100 mg/kg) and pindolol (vehicle only, 1, 3, 10 and 30 mg/kg), respectively. Scans for the same individual rat receiving Wf-516 (n = 4) and pindolol (n = 3) were conducted more than 2 weeks and 1 week apart, respectively. PET imaging was also carried out for rats receiving oral administration of 30 mg/kg fluvoxamine dissolved in 0.5%HPMC 30 min before pindolol treatment in order to investigate the effects of fluvoxamine-induced increase of endogenous 5-HTs on the measurements of 5-HT_1A_ receptor occupancies.

**Figure 8:**
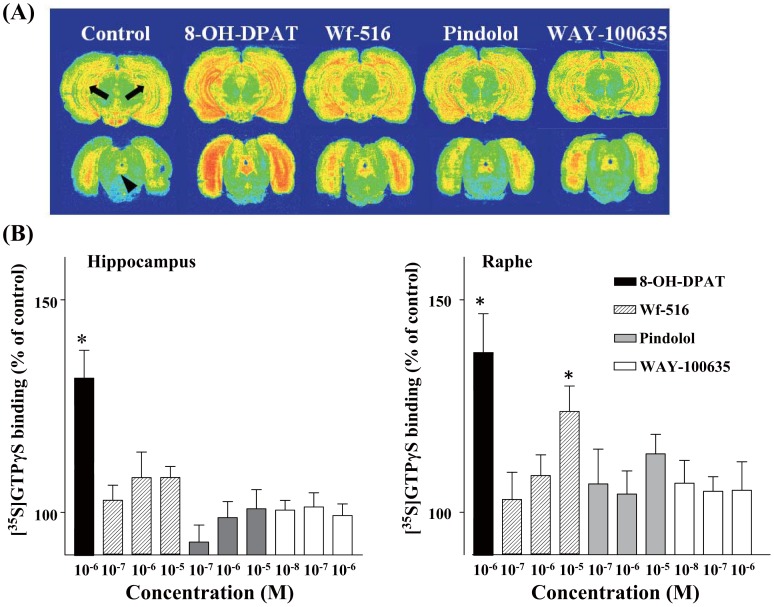
Effects of Wf-516 and pindolol on autoradiographic [^35^S]GTPγS binding in rat brains. (A) Representative autoradiograms showing radiolabeling with [^35^S]GTPγS in the hippocampus and raphe nucleus at baseline (control) or in the presence of 1 µM 8-OH-DPAT (full agonist for 5-HT_1A_ receptors), 10 µM Wf-516, 10 µM pindolol and 1 µM WAY-100635 (full antagonist for 5-HT_1A_ receptors). (B) Ratio of [^35^S]GTPgγS binding to the control level in the hippocampus (left) and raphe nucleus (right). Changes in radiotracer binding were statistically examined using one-way repeated-measures ANOVA followed by least significant difference test. *p<0.01 compared with 1 µM WAY-100635. In the raphe nucleus, a significant interaction between [^35^S]GTPγS binding and three concentrations each of Wf-516, pindolol and WAY-100635 was demonstrated by two-way repeated-measures ANOVA (p<0.05, F(4,20) = 3.58).

All PET scans were carried out using a microPET Focus 220 scanner (Siemens Medical Solutions USA, Knoxville, TN) [27]. The rats were anesthetized with 1.5–2% isoflurane in air (2 L/min flow rate). Emission scans were acquired for 90 min in 3D list mode immediately after intravenous injection of [^11^C]WAY-100635 (at a dose of 106±10 MBq and specific radioactivity of 132±51 GBq/µmol at the time of injection). The injected mass of WAY-100635 ranged from 0.373 to 3.4 nmol, averaging 0.945±0.501 (S.D.) nmol. There were no marked differences in the injected mass of the tracer between the Wf-516 (0.801±0.482 nmol) and pindolol (1.184±0.649 nmol) studies. In the pindolol study, blood samples were collected from the tail vein upon initiation of the scan, and plasma was frozen at −80°C pending assays. All list-mode data were stored into 3D sinograms, which were then Fourier-rebinned into 2D sinograms (26 frames: 4×1, 8×2, and 14×5 min). Images were reconstructed using 2D-filtered back-projection with a 0.5-mm Hanning filter. All PET images were manually coregistered to the MRI template by spatial translation and rotation of original PET images without geometric expansion and contraction. ROIs were placed on the hippocampus and raphe nucleus using PMOD® image analysis software (PMOD Group, Zurich, Switzerland) with reference to the MRI template. Fine-tuning of the location of ROIs was then performed based on PET images at an early phase (0–30 min) of the scan in order to compensate for small mismatches between coregistered PET and MR images. The cerebellum was used as reference tissue because of its negligible density of 5-HT_1A_ receptors [28]. Binding potential based on specific binding compared to nondisplaceable uptake (BP_ND_) for [^11^C]WAY-100635 was quantified by simplified reference tissue model (SRTM), as in a previous clinical PET study using the same radioligand [29]. Occupancies of 5-HT_1A_ receptors by Wf-516 and pindolol were calculated using the following equation:
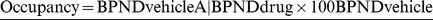
where BP_NDvehicle_ and BP_NDdrug_ are BP_ND_ values in PET analyses after pretreatment with vehicle only and test drug, respectively. The plasma concentration of the test drug needed for 50% occupancy (EC_50_) was determined according to the following relationship:



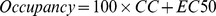
where C is the plasma concentration of pindolol. The ED_50_ value of pindolol was determined as described above.

### [^11^C]WAY-100635-PET Scans of Rats Treated with a Toxicant for 5-HT Neurons

Prior to disruption of 5-HT neurons, rats were scanned using [^11^C]WAY-100635-PET. Central 5-HT neurons were lethally injured by 5,7-DHT as previously described [28]. Briefly, four rats were intraperitoneally pretreated with desipramine (20 mg/kg) to protect noradrenergic neurons, and with pentobarbital (60 mg/kg) 45 min and 10 min before 5,7-DHT treatment, respectively. 5,7-DHT (150 µg as free base in 10 µL of saline containing 0.1% ascorbic acid) was unilaterally infused into the right lateral ventricle (stereotactic coordinates: 0.8 mm anterior to the bregma, 1.2 mm lateral to the midline, and 3.5 mm below the dura mater) for 5 min. More than 2 weeks after 5,7-DHT treatment, a second PET scan was performed to evaluate the extent of the loss of presynaptic 5-HT_1_A receptors. Then, more than 1 week later, a third PET scan was carried out approximately 5 h after oral administration of Wf-516 (30 mg/kg) to the rats. BP_ND_ for 5-HT_1A_ receptors in each PET scan was calculated as described above.

### Measurement of Plasma Pindolol Concentration

(±)-Metoprolol was used as internal standard. Pindolol in the plasma sample was extracted using a solid phase extraction cartridge (OASIS® MCX; Waters, Milford, MA). The eluate was injected into a liquid chromatography-tandem mass spectrometry system equipped with a high performance liquid chromatograph (CLASS-VP HPLC system; Shimadzu, Kyoto, Japan) and a tandem mass spectrometer (TSQ-7000, Thermo Fisher Scientific, San Jose, CA). HPLC analysis was performed on a Xbridge C_18_ column (3.5 µm, 2.1×50 mm; Waters, Milford, MA) at 40°C. The eluted pindolol and (±)-metoprolol were ionized using an electrospray interface and detected by selected reaction monitoring of the transitions of m/z 249.0 to 116.0 and 268.0 to 133.0, respectively.

### Autoradiographic Evaluation of Agonistic Properties of Drugs

Autoradiographic procedures were performed as described previously [30], with some modifications. The sections were preincubated for 15 min in 50 mM 4-(2-hydroxyethyl)-1-piperazineethanesulfonic acid (HEPES; pH 7.4) containing 10 mM NaCl, 3 mM MgCl_2_ and 0.2 mM ethylene glycol tetraacetic acid. A second preincubation using the same buffer containing 2 mM GDP was performed for 15 min. The sections were subsequently incubated in the same buffer containing 2 mM GDP, 0.2 mM dithiothreitol and 0.04 nM [^35^S]GTPγS for 120 min. Basal [^35^S]GTPγS binding was defined in the absence of test drugs. One µM 8-OHDPAT was used as the agonist control, and test drugs included Wf-516 (10^−7^–10^−5^ M), pindolol (10^−7^–10^−5^ M) and WAY-100635 (10^−8^–10^−6^ M). Nonspecific binding was estimated using 10 µM nonradioactive GTPγS. The incubation was terminated by ice-cold 50 mM HEPES (pH 7.0). The sections were desalted with ice-cold distilled water, dried, and placed in contact with an imaging plate for 2 days. Signal detection and image analysis were performed as described above.

## Results

*In vitro* ARG revealed that the inhibition of [^11^C]WAY-100635 binding to 5-HT_1A_ receptors in the hippocampus and raphe nucleus of rats in a manner dependent upon the concentrations of Wf-516 and pindolol in the reaction buffer (Figure 1). Ki values for Wf-516 in the hippocampus and raphe nucleus were 8.1 nM and 7.9 nM, respectively; these values were nearly equivalent to the *in vitro* estimate (7.4 nM) in our previous assay using rat hippocampal homogenates [21]. Pindolol exhibited a slightly higher affinity than Wf-516, as its Ki values in the hippocampus and raphe nucleus were 2.7 nM and 3.6 nM, respectively, also in reasonable agreement with previous *in vitro* ARG measures (7.9 nM and 6.5 nM in the hippocampus and raphe nucleus, respectively) [31]. Excess amounts of Wf-516 and pindolol induced a near-complete blockage of 5-HT_1A_ receptors, and there were no differences in their affinities between the hippocampus and raphe nucleus [p>0.05 by two-way repeated-measures analysis of variance (ANOVA)]. The lack of regional selectivity of Wf-516 and pindolol was also demonstrated by *ex vivo* ARG analyses (Figure 2), in which Wf-516 and pindolol bound to 5-HT_1A_ receptors in a dose-dependent manner up to approximately 80% and 70%, respectively, irrespective of the region. Oral ED_50_ values for Wf-516 in the hippocampus and raphe nucleus were 5.3 mg/kg and 4.2 mg/kg, respectively, and intraperitoneal ED_50_ values for pindolol in these regions were 8.5 mg/kg and 7.3 mg/kg, respectively. There was no significant difference in drug occupancies between the hippocampus and raphe nucleus (p>0.05 by two-way repeated-measures ANOVA).

In contrast to *in vitro* and *ex vivo* autoradiographic imaging, the *in vivo* PET assays indicated a limited capacity of 5-HT_1A_ receptors to be accessible to Wf-516, with a marked regional difference. The partial and full inhibitions of [^11^C]WAY-100635 binding by pretreated Wf-516 and pindolol, respectively, were visually demonstrated in representative PET images showing dose-dependent changes in the same individual rats (Figure 3). Effects of Wf-516 and pindolol on the radioligand kinetics in the brain were then quantitatively assessed by defining ROIs on dynamic PET data. Time-radioactivity curves after administration of [^11^C]WAY-100635 demonstrated that the reduction of radioligand retention with increasing doses of pretreated Wf-516 was more prominent in the raphe nucleus than in the hippocampus (Figure 4). However, time-radioactivity curves in these regions showed a substantial difference from that in the cerebellum even with an excessive dose of this drug. Meanwhile, [^11^C]WAY-100635 radiosignals in the hippocampus and raphe nucleus were attenuated to a level close to cerebellar values as a function of the dose of pretreated pindolol (Figure 4). These distinct properties in the mode of receptor occupancies by Wf-516 and pindolol were more clearly presented by plotting the specific binding of [^11^C]WAY-100635 calculated as the difference in radioactivity between target and reference regions (Figure 5). Again, an incomplete but regionally selective suppression of radioligand binding following Wf-516 pretreatment was demonstrated, while these binding components were fully displaceable by pindolol.

We then quantified BP_ND_ values for radioligand binding and occupancies of 5-HT_1A_ receptors by Wf-516 and pindolol at each dose (Table 1 and Figure 6). Both drugs induced attenuation of BP_ND_ in a dose-dependent fashion. Notably, Wf-516 preferentially bound to 5-HT_1A_ receptors in the raphe nucleus compared to the hippocampus. This regional selectivity was particularly striking at a high dose, and a statistically significant interaction between Wf-516 dose and region was observed (p<0.01, F(4,12) = 7.53 by two-way repeated-measures ANOVA). Saturation occupancy by Wf-516 was far below 100%, but there was a more than 2.5-fold difference in maximal occupancy as calculated by the regression curve between the hippocampus (20.7%) and raphe nucleus (53.8%). In contrast, the dose-dependent binding of pindolol to 5-HT_1A_ receptors reached nearly full occupancy in these regions. A significant main effect of region on receptor occupancies by pindolol was found (p<0.01, F(1,2) = 157.6 by two-way repeated-measures ANOVA), indicating that this drug also binds to 5-HT_1A_ receptors in a somewhat regionally selective manner, although there was no marked interaction between dose and region (p>0.05 by two-way repeated-measures ANOVA). Curve fitting in scatterplots of 5-HT_1A_ receptor occupancy against dose and plasma concentration of pindolol yielded the ED_50_ and EC_50_ values for this drug, respectively (Figure 6). ED_50_ estimates in the hippocampus and raphe nucleus were 5.6 mg/kg and 2.4 mg/kg, respectively, and EC_50_ measures in these regions were 319 ng/mL and 125 ng/mL, respectively. The occupancy by 3 mg/kg Wf-516, corresponding to ED_50_ for its blockade of 5-HTTs as reported in our previous study [24], differed by approximately 100% between the hippocampus and raphe nucleus, while 3 mg/kg pindolol, close to its ED_50_ value in the raphe nucleus, resulted in an approximately 50% difference in the occupancy between these two regions, suggesting a prominent regional selectivity of Wf-516 in comparison with pindolol. Additionally, administration of 30 mg/kg fluvoxamine prior to pindolol treatment, which is capable of inhibiting more than 80% of 5-HTTs according to our previous investigation [24], had no effect on 5-HT_1A_ receptor occupancies by pindolol (Table 1). This result implies that binding of 5-HT_1A_ receptor ligands was not overtly affected by a concurrent blockade of 5-HTT and consequent increase of synaptic 5-HT, supporting the view that the characteristics of the interaction between Wf-516 and 5-HT_1A_ receptors do not stem from its dual action on 5-HT_1A_ receptor and 5-HTT.

As the preferential binding of Wf-516 to the raphe nucleus could be due to its selectivity for presynaptic 5-HT_1A_ autoreceptors enriched in this region, we assessed the occupancy of 5-HT_1A_ receptors by Wf-516 in rats treated with 5,7-DHT, which abolished 5-HT neurons expressing presynaptic 5-HT_1A_ receptors. Consistent with our previous *ex vivo* study [28], BP_ND_ for [^11^C]WAY-100635 in the hippocampus was almost unchanged by treatment with 5,7-DHT, while that in the raphe nucleus was profoundly decreased, indicating a selective disruption of 5-HT neurons bearing presynaptic 5-HT_1A_ receptors (Figure 7). The residual radioligand binding in the raphe nucleus after treatment with 5,7-DHT was presumed to primarily reflect the presence of binding sites other than presynaptic 5-HT_1A_ autoreceptors, as documented in a previous study [32]. Oral administration of 30 mg/kg Wf-516 to these 5,7-DHT-treated rats induced a significant decrease of BP_ND_ in the hippocampus as compared with baseline, but no additional reduction of BP_ND_ was observed in the raphe nucleus.

We subsequently investigated the agonistic and antagonistic properties of Wf-516, in consideration of the fact that several drugs acting as partial agonists for 5-HT_1A_ receptor display a high selectivity for presynaptic binding sites relative to postsynaptic components [33]–[35]. *In vitro* ARG binding of [^35^S]GTPγS was enhanced by the addition of a 5-HT_1A_ receptor agonist, 8-OH-DPAT, in the hippocampus and raphe nucleus (Figure 8). In the raphe nucleus, a significant interaction between [^35^S]GTPγS binding and three different concentrations of each of Wf-516, pindolol and WAY-100635 was observed (p<0.05, F(4,20)  = 3.58 by two-way repeated-measures ANOVA). Furthermore, 1 µM 8-OH-DPAT and 10 µM Wf-516 significantly increased [^35^S]GTPγS binding in the raphe nucleus as compared with 1 µM WAY-100635 (p<0.01 by one-way repeated-measures ANOVA followed by least significant difference test), with the increment by Wf-516 being smaller than that by 8-OH-DPAT. The increased binding of [^35^S]GTPγS by Wf-516 was abolished in the presence of 1 µM 8-OH-DPAT (data not shown). In the hippocampus, test compounds other than 8-OH-DPAT did not induce a significant increase in [^35^S]GTPγS binding (p>0.05 by one-way repeated-measures ANOVA followed by least significant difference test), although there was a tendency for enhancement of radioprobe binding by Wf-516 at higher concentrations. Pindolol at high concentrations also produced a slight increase in [^35^S]GTPγS binding in the hippocampus and raphe nucleus, but this change was not statistically significant (p>0.05 by one-way repeated-measures ANOVA followed by least significant difference test). These experimental data suggest a partial agonist action of Wf-516 on 5-HT_1A_ receptors, which may be relevant to its binding selectivity for presynaptic elements.

## Discussion

In this study, we demonstrated preferential binding of Wf-516 to presynaptic 5-HT_1A_ autoreceptors by means of *in vivo* PET imaging of rat brains. This investigational drug dually acting on 5-HTTs and 5-HT_1A_ receptors yielded approximately 20% and 50% occupancies at maximum in the hippocampus and raphe nucleus of living rats, respectively. PET experiments following toxic injuries of 5-HT neurons by 5,7-DHT indicated a selective affinity of Wf-516 for presynaptic receptors accounting for this regionality. The limited availability of 5-HT_1A_ receptors to Wf-516 and its regional difference was in sharp contrast with an established 5-HT_1A_ antagonist, pindolol, which fully occupied these receptors at a high dose irrespective of region. Since one of the major differences in pharmacological features between Wf-516 and pindolol is the blockage of 5-HTTs, it was initially presumed that synaptic 5-HT, intensified by the inhibitory effects of Wf-516 on 5-HTTs, could in turn compete with Wf-516 for 5-HT_1A_ receptors, resulting in limited receptor occupancy by Wf-516. However, this possibility was ruled out by our PET observation that the receptor occupancies by pindolol were apparently not influenced by co-treatment with a typical SSRI, fluvoxamine, at a dose of 30 mg/kg, supposedly blocking 80% of 5-HTTs [24]. Furthermore, these observations are not likely to result from partial volume effects in the raphe ROI, which could lead to underestimation of BP_ND_ but not overestimation of the receptor occupancy by Wf-516. Indeed, we defined a relatively large brainstem ROI including the raphe and surrounding structures for increasing the signal-to-noise ratio, and therefore the receptor occupancy might be somewhat underestimated due to the presence of nonspecific signals from non-raphe areas, which were not displaceable by Wf-516. Despite this possible effect, the occupancy by Wf-516 in the raphe nucleus was higher than that in the hippocampus. Another possibility for the regional difference in the receptor occupancy by Wf-516 is the variability of its uptake among brain areas attributed to locally differential effects of efflux transporters. However, this is also unlikely in light of: 1) efficient transfer of Wf-516 to the brain with the brain-to-plasma ratio approximating 2.0 at the time of reaching maximal plasma concentration (unpublished data); and 2) our previous observation that the occupancy of 5-HTT by Wf-516 was homogenous among regions [24].

We then postulated that Wf-516 might exert agonistic effects at 5-HT_1A_ receptors, in view of previous clinical studies documenting that occupancies of 5-HT_1A_ receptors by agonistic agents at regular doses without adverse effects were barely detectable by [^11^C]WAY-100635-PET [36]–[38]. This notion was supported by the present ARG measurements of [^35^S]GTPγS, which indicated the partial agonistic potency of Wf-516, particularly in the raphe nucleus. Since it was reported that 5-HT_1A_ receptors were configured in high- and low-affinity states for agonistic ligands [39], the incomplete occupancy of these receptors by Wf-516 can be explained by its selectivity for the high-affinity state binding components. Agonists are supposed to interact with G protein-coupled and uncoupled 5-HT_1A_ receptors with high and low affinities, respectively, and these two modes of the receptors may coexist in the same synaptic terminal. Previous *in vitro* ligand-receptor binding assays also indicated two such distinct functional modes in other serotonin receptor subtypes, including 5-HT_1B_ [40], 5-HT_2A_ [41], [42] and 5-HT_2C_ [42] receptors, and G protein-coupled neurorecptors in non-serotonergic systems [43]. The proportion of the high-affinity sites against total binding components might differ between presynaptic and postsynaptic terminals, which might be associated with the previous observation that 5-HT_1A_ receptor reserve for agonists in the hippocampus was lower than that in the dorsal raphe nucleus [44], [45]. An *in vivo* electrophysiological study of rats demonstrated that Wf-516 intravenously administered at low and medium doses inhibited the actions of 5-HT_1A_ autoreceptor agonists on firing of 5-HT neurons, but enhanced these agonistic effects at high cumulative doses [23]. This supports the contention that Wf-516 preferentially binds to agonist 5-HT_1A_ receptor sites, acts antagonistically at therapeutic doses, and exerts an agonistic property at excessive doses. The previous finding that Wf-516 at low and medium doses is devoid of activity in hippocampal postsynaptic 5-HT_1A_ receptors [23] is also consistent with the fact that postsynaptic receptors are relatively insensitive to partial agonists [33]–[35]. Despite these indications, it is still necessary to assess differences in the density of agonist binding sites in the raphe nucleus and hippocampus of living brains using PET and agonistic radioligands for 5-HT_1A_ receptors. Such imaging agents have recently been developed [46], [47] and would serve this purpose.

Unlike Wf-516, pindolol at a high dose showed full occupancy of 5-HT_1A_ receptors in the hippocampus and raphe nucleus, while binding of this compound at low and medium doses appeared somewhat regionally selective. This modest regionality of receptor occupancies by pindolol may account for inconsistent observations in clinical PET investigations of its binding selectivity [16]–[18]. Accordingly, the present ARG using [^35^S]GTPγS suggested marginal, insignificant agonistic activity of pindolol at 5-HT_1A_ receptors. This conflicts slightly with a previous study that showed unremarkable modulations of ARG [^35^S]GTPγS binding by pindolol [30], but is basically in agreement with reports demonstrating the *in vivo* electrophysiological effects of pindolol as an agonist [48] and its mixed antagonistic/agonistic profile in physiological experiments [49]. Considering these observations together with the inconsistency of previous reports on the selectivity of pindolol for presynaptic sites [16]–[20], we postulate that pindolol does not strongly preferentially interact with high-affinity binding sites for agonists and therefore may not be as suitable as Wf-516 for selectively blocking and/or downregulating presynaptic 5-HT_1A_ autoreceptors.

As illustrated in Figure 6C, EC_50_ estimates for receptor occupancies by pindolol in the raphe nucleus and hippocampus were 125 and 319 ng/mL, respectively, which were not equivalent to the clinical data (27 and 80 ng/mL in the raphe nucleus and postsynaptic receptor-rich region, respectively) of one previous PET study [16], but they were acceptably close to one another to support the predictability of clinical doses based on animal measures. Because the proportions of protein-bound pindolol in plasma or serum have been reported to be 52% in rats [50] and 57% in humans [51], and because no metabolites of this drug active at 5-HT_1A_ receptors have been identified, we assumed that there were no species differences in the relationships between plasma pindolol concentration and 5-HT_1A_ receptor occupancy. In clinical assays, data on the plasma concentration of pindolol to induce full occupancy of receptors are lacking due to concerns about side effects at such high doses, and this may hamper accurate determination of EC_50_ in humans, conceivably resulting in a slight difference in EC_50_ values between non-clinical and clinical studies. Indeed, pindolol was required to fully block 5-HT_1A_ autoreceptors in order to provide benefits for the therapeutic effects of SSRIs, but it was found to occupy only 60% of these receptors at the maximal safe dose [16]. As illustrated by our PET measurements, the high-dose administration of pindolol sufficient for the full occupancy of 5-HT_1A_ autoreceptors in the raphe nucleus simultaneously causes a complete blockade of postsynaptic receptors in the hippocampus, a crucial drawback for the antidepressive effects of SSRIs mediated directly by reinforcement of serotonergic neurotransmissions and indirectly by the resultant enhancement of neurogenesis [52], [53]. This could be in line with the results of clinical studies [54], [55] that failed to confirm the adjunctive therapeutic effects of pindolol reported in earlier works [11].

Besides the utilization of common methodologies and biological parameters among species to facilitate the translation of findings from non-clinical to clinical studies, *in vivo* PET imaging has the advantage of being able to clarify the status of bioactive molecules in living brains, which might not be revealed by *in vitro* or *ex vivo* techniques. In fact, the pharmacokinetic characteristics of Wf-516, including limited ranges of 5-HT_1A_ receptor occupancy and preference of presynaptic receptors, were demonstrated by PET but not by other assaying modalities. Although the reasons for these discrepant observations among analytical methods remain unclear, one could speculate that alterations in 5-HT_1A_ receptors – including their subcellular localizations, biochemical modifications (e.g., phosphorylation, glycosylation), coupling to G proteins, and proportion of high-affinity sites, in addition to redistribution of the drug – could occur at perimortem and postmortem periods in the preparations of *in vitro* and *ex vivo* samples. Indeed, *ex vivo* receptor occupancy measurements in the present work were conducted by reacting radioligands with brain samples collected from Wf-516- or pindolol-treated rats, and thus could be influenced by perimortem and postmortem alterations of the receptor statuses, unlike *ex vivo* autoradiographic labeling of the receptors with radioligands systemically administered to living animals. This discrepancy between *in vivo* and postmortem assays may be relevant to a previous observation that binding of an agonistic radioligand for 5-HT_1A_ receptors in the cat hippocampus was abundant in autoradiograms but was nearly absent in PET images [47]. One speculative notion would be that recoupling of low-affinity-state receptors to G proteins to form a high-affinity state occurs at a higher rate in perimortem and postmortem conditions than in living brains, and this could facilitate interaction between agonists and receptors in *ex vivo* and *in vitro* autoradiographic assays. Transition between high- and low-affinity states was also implied in dopamine D_2_ receptors [56], although the molecular mechanisms by which neuroreceptors are coupled to and uncoupled from G proteins have yet to be clarified.

The present data also provide mechanistic implications for other in-development drugs simultaneously targeting 5-HTT and 5-HT_1A_ receptors, such as vilazodone (also known as EMD68843) [57]. This drug has been developed as an SRI as well as a partial agonist for 5-HT_1A_ receptors, but *in vivo* biochemical assays in animals have supported its activity as an antagonist for these receptors [58]. This is quite similar to observations in a previous electrophysiological experiment using Wf-516 at low and medium doses [23]. Moreover, a clinical PET study with [^11^C]WAY-100635 implied that occupancies of 5-HT_1A_ receptors by vilazodone exhibited selectivity for presynaptic components and did not reach a complete blockade of these receptors, despite its small sample size [38]. The properties resembling those of Wf-516 can be more precisely assessed by small animal PET measurements, and each subject in such non-clinical studies can be repeatedly scanned after administration of drugs at different doses broadly ranging from minimal to excessive amounts, an approach not possible for human subjects. As vilazodone has been proven to be effective in treating patients with major depression in a phase III clinical trial [59] and has lately been approved by the US Food and Drug Administration [60], our results have brought to light evidence of promising clinical potencies of Wf-516.

In conclusion, the present results, in conjunction with our previous data [24], have proven the pharmacological concept of an emerging antidepressant therapy dually targeting the 5-HTT and presynaptic 5-HT_1A_ receptors. This combination enables prompt serotonergic enhancement without autoreceptor-mediated negative feedback. The partial agonistic properties of Wf-516 could be linked to the presynaptic dominance of the drug action, and would thus be suitable for inhibitory blockade of presynaptic signaling suppressive of 5-HT release.

## References

[1] Hamed A, Lee A, Ren XS, Miller DR, Cunningham F, et al (2004) Use of antidepressant medications: are there differences in psychiatric visits among patient treatments in the Veterans Administration? Med Care 42: 551–559. 15167323 10.1097/01.mlr.0000128002.73998.87

[2] Hansen DG, Søndergaard J, Vach W, Gram LF, Rosholm JU, et al (2003) Antidepressant drug use in general practice: inter-practice variation and association with practice characteristics. Eur J Clin Pharmacol 59: 143–149. 12721774 10.1007/s00228-003-0593-3

[3] Uchida N, Chong MY, Tan CH, Nagai H, Tanaka M, et al (2007) International study on antidepressant prescription pattern at 20 teaching hospitals and major psychiatric institutions in East Asia: Analysis of 1898 cases from China, Japan, Korea, Singapore and Taiwan. Psychiatry Clin Neurosci 61: 522–528. 17875031 10.1111/j.1440-1819.2007.01702.x

[4] MacGillivray S, Arroll B, Hatcher S, Ogston S, Reid I, et al (2003) Efficacy and tolerability of selective serotonin reuptake inhibitors compared with tricyclic antidepressants in depression treated in primary care: systematic review and meta-analysis. BMJ 326: 1014–1017. 12742924 10.1136/bmj.326.7397.1014 PMC154760

[5] Hyttel J (1994) Pharmacological characterization of selective serotonin reuptake inhibitors (SSRIs). Int Clin Psychopharmacol Suppl 1: 19–26. 10.1097/00004850-199403001-00004 8021435

[6] Adell A, Artigas F (1991) Differential effects of clomipramine given locally or systemically on extracellular 5-hydroxytryptamine in raphe nuclei and frontal cortex. An in vivo brain microdialysis study. Naunyn Schmiedebergs Arch Pharmacol 343: 237–244. 1714040 10.1007/BF00251121

[7] Invernizzi R, Belli S, Samanin R (1992) Citalopram’s ability to increase the extracellular concentrations of serotonin in the dorsal raphe prevents the drug’s effect in the frontal cortex. Brain Res 584, 322–324. 10.1016/0006-8993(92)90914-u 1515949

[8] Chaput Y, de Montigny C, Blier P (1991) Presynaptic and postsynaptic modifications of the serotonin system by long-term administration of antidepressant treatments. An in vivo electrophysiologic study in the rat. Neuropsychopharmacology 5, 219–229. 1839498

[9] Hjorth S, Auerbach SB (1994) Further evidence for the importance of 5-HT_1A_ autoreceptors in the action of selective serotonin reuptake inhibitors. Eur J Pharmacol 260: 251–255. 7988652 10.1016/0014-2999(94)90346-8

[10] Celada P, Puig M, Amargós-Bosch M, Adell A, Artigas F (2004) The therapeutic role of 5-HT_1A_ and 5-HT_2A_ receptors in depression. J Psychiatry Neurosci 29: 252–265. 15309042 PMC446220

[11] Artigas F, Romero L, de Montigny C, Blier P (1996) Acceleration of the effect of selected antidepressant drugs in major depression by 5-HT_1A_ antagonists. Trends Neurosci 19: 378–383. 8873352 10.1016/S0166-2236(96)10037-0

[12] Pérez V, Gilaberte I, Faries D, Alvarez E, Artigas F (1997) Randomised, double-blind, placebo-controlled trial of pindolol in combination with fluoxetine antidepressant treatment. Lancet 349: 1594–1597. 9174562 10.1016/S0140-6736(96)08007-5

[13] Zanardi R, Artigas F, Franchini L, Sforzini L, Gasperini M, et al (1997) How long should pindolol be associated with paroxetine to improve the antidepressant response? J Clin Psychopharmacol 17: 446–450. 9408806 10.1097/00004714-199712000-00002

[14] Zanardi R, Franchini L, Gasperini M, Lucca A, Smeraldi E, et al (1998) Faster onset of action of fluvoxamine in combination with pindolol in the treatment of delusional depression: a controlled study. J Clin Psychopharmacol 18: 441–446. 9864075 10.1097/00004714-199812000-00004

[15] Pike VW, McCarron JA, Lammertsma AA, Osman S, Hume SP, et al (1996) Exquisite delineation of 5-HT1A receptors in human brain with PET and [carbonyl-11 C]WAY-100635. Eur J Pharmacol 301: R5–7. 8773468 10.1016/0014-2999(96)00079-9

[16] Martinez D, Hwang D, Mawlawi O, Slifstein M, Kent J, et al (2001) Differential occupancy of somatodendritic and postsynaptic 5-HT_1A_ receptors by pindolol: a dose-occupancy study with [^11^C]WAY 100635 and positron emission tomography in humans. Neuropsychopharmacology 24: 209–229. 11166513 10.1016/S0893-133X(00)00187-1

[17] Rabiner EA, Gunn RN, Castro ME, Sargent PA, Cowen PJ, et al (2000) Beta-blocker binding to human 5-HT_1A_ receptors in vivo and in vitro: implications for antidepressant therapy. Neuropsychopharmacology 23: 285–293. 10942852 10.1016/S0893-133X(00)00109-3

[18] Andrée B, Thorberg SO, Halldin C, Farde L (1999) Pindolol binding to 5-HT_1A_ receptors in the human brain confirmed with positron emission tomography. Psychopharmacology (Berlin) 144: 303–305. 10435400 10.1007/s002130051009

[19] Hirani E, Opacka-Juffry J, Gunn R, Khan I, Sharp T, et al (2000) Pindolol occupancy of 5-HT_1A_ receptors measured in vivo using small animal positron emission tomography with carbon-11 labeled WAY 100635. Synapse 36: 330–341. 10819911 10.1002/(SICI)1098-2396(20000615)36:4<330::AID-SYN10>3.0.CO;2-H

[20] Corradetti R, Laaris N, Hanoun N, Laporte AM, Le Poul E, et al (1998) Antagonist properties of (-)-pindolol and WAY 100635 at somatodendritic and postsynaptic 5-HT_1A_ receptors in the rat brain. Br J Pharmcol 123: 449–462. 10.1038/sj.bjp.0701632 PMC1565192 9504386

[21] Maeda J, Morio Y, Oohashi Y, Katayama J, Nishiyama A, et al (2006) Wf-516, a novel antidepressant with dual serotonergic activity: l. Receptorial and pharmacological profiles in comparison with reference antidepressants. European Neuropsychopharmacol 16: S333.

[22] Papp M, Gruca P, Litwa E, Lason M, Przegalinski E (2006) Antidepressant-like activity of Wf-516, an antagonist of 5-HT_1A_ receptors and inhibitor of 5-HT reuptake, in a chronic mild stress model of depression in rat. Eur Neuropsychopharmacol 16: S305–S306.

[23] El Mansari M, Blier P (2008) In vivo electrophysiological assessment of the putative antidepressant Wf-516 in the rat raphe dorsalis, locus coeruleus and hippocampus. *Naunyn Schmiedebergs Arch Pharmacol* 376: 351–361. 18060386 10.1007/s00210-007-0210-6

[24] Saijo T, Maeda J, Okauchi T, Maeda J-i, Morio Y, et al (2009) Utility of small animal positron emission tomographic imaging of rats for preclinical development of drugs acting on serotonin transporter. Int J Neuropsychopharmacol 12: 1021–1032. 19236731 10.1017/S1461145709000042

[25] McCarron JA, Turton DR, Pike VW, Polle KG (1996) Remotely-controlled production of the 5-HT_1A_ radioligand, [carbonyl-^11^C]WAY-100635, via ^11^C-carbokylation of an immobilized Grignard reagent. J Label Compd Radiopharm 38: 941–953.

[26] Khawaja X, Evans N, Reilly Y, Ennis C, Minchin MC (1995) Characterization of the binding of [^3^H]WAY-100635, a novel 5-hydroxytryptamine1A receptor antagonist, to rat brain. J Neurochem 64: 2716–2726. 7760052 10.1046/j.1471-4159.1995.64062716.x

[27] Tai YC, Ruangma A, Rowland D, Siegel S, Newport DF, et al (2005) Performance evaluation of the microPET focus: A third-generation microPET scanner dedicated to animal imaging. J Nucl Med 46: 455–463. 15750159

[28] Maeda J, Suhara T, Ogawa M, Okauchi T, Kawabe K, et al (2001) In vivo binding properties of [carbonyl-^11^C]WAY-100635: effect of endogenous serotonin. Synapse 40: 122–129. 11252023 10.1002/syn.1033

[29] Takano A, Ito H, Arakawa R, Saijo T, Suhara T (2007) Effects of the reference tissue setting on the parametric image of ^11^C-WAY 100635. Nucl Med Commun 28, 193–198. 10.1097/MNM.0b013e328013ebec 17264778

[30] Serrats J, Artigas F, Mengod G, Cortés R (2004) An autoradiographic study of the influence of pindolol upon [^35^S]GTPγS binding in rat, guinea pig and human brain. Int J Neuropsychopharmacol 7: 27–34. 14720318 10.1017/S1461145703003924

[31] Castro ME, Harrison PJ, Pazos A, Sharp T (2000) Affinity of (+/−)-pindolol, (-)-penbutolol, and (-)-tertatolol for pre- and postsynaptic serotonin 5-HT_1A_ receptors in human and rat brain. J Neurochem 75: 755–762. 10899952 10.1046/j.1471-4159.2000.0750755.x

[32] Kirby LG, Pernar L, Valentino RJ, Beck SG (2003) Distinguishing characteristics of serotonin and non-serotonin-containing cells in the dorsal raphe nucleus: electrophysiological and immunohistochemical studies. Neuroscience 116: 669–683. 12573710 10.1016/s0306-4522(02)00584-5 PMC2832757

[33] Andrade R, Nicoll RA (1987) Pharmacologically distinct actions of serotonin on single pyramidal neurones of the rat hippocampus recorded in vitro. J Physiol 394: 99–124. 3443977 10.1113/jphysiol.1987.sp016862 PMC1191953

[34] Van den Hooff P, Galvan M (1992) Actions of 5-hydroxytryptamine and 5-HT_1A_ receptor ligands on rat dorso-lateral septal neurones in vitro. Br J Pharmacol 106: 893–899. 1393288 10.1111/j.1476-5381.1992.tb14431.x PMC1907649

[35] Van den Hoof P, Galvan M (1991) Electrophysiology of the 5-HT_1A_ ligand MDL 73005EF in the rat hippocampal slice. Eur J Pharmacol 196: 291–298. 1893914 10.1016/0014-2999(91)90442-s

[36] Bantick RA, Rabiner EA, Hirani E, de Vries MH, Hume SP, et al (2004) Occupancy of agonist drugs at the 5-HT_1A_ receptor. Neuropsychopharmacology 29: 847–859. 14985704 10.1038/sj.npp.1300390

[37] Nakayama T, Suhara T, Okubo Y, Ichimiya T, Yasuno F, et al (2002) In vivo drug action of tandospirone at 5-HT_1A_ receptor examined using positron emission tomography and neuroendocrine response. Psychopharmacology (Berlin) 165: 37–42. 12474116 10.1007/s00213-002-1234-8

[38] Rabiner EA, Gunn RN, Wilkins MR, Sargent PA, Mocaer E, et al (2000) Drug action at the 5-HT_1A_ receptor in vivo: Autoreceptor and postsynaptic receptor occupancy examined with PET and [carbonyl-^11^C]WAY-100635. Nucl Med Biol 27: 509–513. 10962259 10.1016/s0969-8051(00)00120-7

[39] Assie MB, Cosi C, Koek W (1999) Correlation between low/high affinity ratios for 5-HT_1A_ receptors and intrinsic activity. Eur J Pharmacol 386: 97–103. 10611469 10.1016/s0014-2999(99)00738-4

[40] Ng GY, George SR, Zastawny RL, Caron M, Bouvier M, et al (1993) Human serotonin1B receptor expression in Sf9 cells: phosphorylation, palmitoylation, and adenylyl cyclase inhibition. Biochemistry 32: 11727–11733. 8218242 10.1021/bi00094a032

[41] Herndon JL, Glennon RA (1993) Serotonin receptors, agents, and actions. In: Kozikowsky A, editor. Drug design, molecular modeling, and the neurosciences. New York: Raven Press. Pp. 167–212.

[42] Fitzgerald LW, Conklin DS, Krause CM, Marshall AP, Patterson JP, et al (1999) High-affinity agonist binding correlates with efficacy (intrinsic activity) at the human serotonin 5-HT_2A_ and 5-HT_2C_ receptors: evidence favoring the ternary complex and two-state models of agonist action. J Neurochem 72: 2127–2134. 10217294 10.1046/j.1471-4159.1999.0722127.x

[43] Finnema SJ, Bang-Andersen B, Wikström HV, Halldin C (2010) Current state of agonist radioligands for imaging of brain dopamine D2/D3 receptors in vivo with positron emission tomography. Curr Top Med Chem 10: 1477–1498. 20583987 10.2174/156802610793176837

[44] Meller E, Goldstein M, Bohmaker K (1990) Receptor reserve for 5-hydroxytryptamine1A-mediated inhibition of serotonin synthesis: possible relationship to anxiolytic properties of 5-hydroxytryptamine1A agonists. Mol Pharmacol 37: 231–237. 1968223

[45] Yocca FD, Iben L, Meller E (1992) Lack of apparent receptor reserve at postsynaptic 5-hydroxytryptamine1A receptors negatively coupled to adenylyl cyclase activity in rat hippocampal membranes. Mol Pharmacol 41: 1066–1072. 1352034

[46] Milak MS, Severance AJ, Ogden RT, Prabhakaran J, Kumar JS, et al (2008) Modeling considerations for ^11^C-CUMI-101, an agonist radiotracer for imaging serotonin 1A receptor in vivo with PET. J Nucl Med 49: 587–596. 18344443 10.2967/jnumed.107.046540 PMC3580231

[47] Lemoine L, Verdurand M, Vacher B, Blanc E, Le Bars D, et al (2010) [^18^F]F15599, a novel 5-HT_1A_ receptor agonist, as a radioligand for PET neuroimaging. Eur J Nucl Med Mol Imaging 37: 594–605. 19789870 10.1007/s00259-009-1274-y

[48] Sprouse J, Braselton J, Reynolds L (2000) 5-HT_1A_ agonist potential of pindolol: electrophysiologic studies in the dorsal raphe nucleus and hippocampus. Biol Psychiatry 47: 1050–1055. 10862804 10.1016/s0006-3223(99)00322-4

[49] Sánchez C, Arnt J, Moltzen E (1996) Assessment of relative efficacies of 5-HT_1A_ receptor ligands by means of in vivo animal models. Eur J Pharmacol 315: 245–254. 8982661 10.1016/s0014-2999(96)00621-8

[50] Komori T, Shimoishi K, Harashima H, Otagiri M (1998) Effects of repeated clarithromycin administration on the pharmacokinetic properties of pindolol in rats. Biol Pharm Bull 21: 1376–1378. 9881659 10.1248/bpb.21.1376

[51] Gugler R, Herold W, Dengler HJ (1974) Pharmacokinetics of pindolol in man. Eur J Clin Pharmacol 7: 17–24. 4853602 10.1007/BF00614385

[52] Duman RS, Malberg J, Nakagawa S (2001) Regulation of adult neurogenesis by psychotropic drugs and stress. J Pharmacol Exp Ther 299: 401–407. 11602648

[53] Radley JJ, Jacobs BL (2002) 5-HT_1A_ receptor antagonist administration decreases cell proliferation in the dentate gyrus. Brain Res 955: 264–267. 12419546 10.1016/s0006-8993(02)03477-7

[54] Berman RM, Darnell AM, Miller HL, Anand A, Charney DS (1997) Effect of pindolol in hastening response to fluoxetine in the treatment of major depression: a double-blind, placebo-controlled trial. Am J Psychiatry 154: 37–43. 8988956 10.1176/ajp.154.1.37

[55] McAskill R, Mir S, Taylor D (1998) Pindolol augmentation of antidepressant therapy. Br J Psychiatry 173: 203–208. 9926094 10.1192/bjp.173.3.203

[56] Seeman P (2012) Dopamine agonist radioligand binds to both D2High and D2Low receptors, explaining why alterations in D2High are not detected in human brain scans. Synapse 66: 88–93. 21954082 10.1002/syn.20987

[57] Dawson LA, Watson JM (2009) Vilazodone: a 5-HT_1A_ receptor agonist/serotonin transporter inhibitor for the treatment of affective disorders. CNS Neurosci Ther 15: 107–117. 19499624 10.1111/j.1755-5949.2008.00067.x PMC6493994

[58] Hughes ZA, Starr KR, Langmead CJ, Hill M, Bartoszyk GD, et al (2005) Neurochemical evaluation of the novel 5-HT_1A_ receptor partial agonist/serotonin reuptake inhibitor, vilazodone. Eur J Pharmacol 510: 49–57. 15740724 10.1016/j.ejphar.2005.01.018

[59] Rickels K, Athanasiou M, Robinson DS, Gibertini M, Whalen H, et al (2009) Evidence for efficacy and tolerability of vilazodone in the treatment of major depressive disorder: a randomized, double-blind, placebo-controlled trial. J Clin Psychiatry 70: 326–333. 19284933 10.4088/jcp.08m04637

[60] Laughren TP, Gobburu J, Temple RJ, Unger EF, Bhattaram A, et al (2011) Vilazodone: clinical basis for the US Food and Drug Administration’s approval of a new antidepressant. J Clin Psychiatry 72: 1166–1173. 21951984 10.4088/JCP.11r06984

